# Supplementary material for: EnzML: multi-label prediction of enzyme classes using InterPro signatures
Source: BMC Bioinformatics. 2012 Apr 25;13:61. doi: 10.1186/1471-2105-13-61 (PMC3483700; doi:10.1186/1471-2105-13-61)
Supplement: Addtional file 5 — The Java code to format the data files, evaluate and predict. The file enzml_java_code.tar.gz contains the Java code used to format database data to ARFF and XML formats, to execute cross and train-test (jackknife) evaluations and to record evaluation results to database. More information is included in the readme.txt file and the Javadoc files. The code can be used with a MySQL database. To use a different database software, other JDBC drivers might be required. [file 1471-2105-13-61-S5.gz › java_code/utils/doc/uk/ac/ed/inf/utils/maputils/TableMap.html]

TableMap


---


|  |  |  |
| --- | --- | --- |
| **Overview** \| **Package** \| **Class** \| **Use** \| **Tree** \| **Deprecated** \| **Index** \| **Help** | |  |
| **PREV CLASS**   NEXT CLASS | **FRAMES**    **NO FRAMES**     **All Classes** |  |
| SUMMARY: NESTED \| FIELD \| CONSTR \| METHOD | DETAIL: FIELD \| CONSTR \| METHOD |  |


---


## uk.ac.ed.inf.utils.maputils Class TableMap

```
java.lang.Object
  uk.ac.ed.inf.utils.maputils.TableMap
```

---

``` public class TableMap extends java.lang.Object ```

A table map is a map containing relations [key > list of values] [value >
list of keys]

**Version:**
:   3 Jun 2008

**Author:**
:   Luna De Ferrari luna.deferrari-at-ed.ac.uk

---

**Constructor Summary**

`TableMap()`
  
          Initialises the object with an empty 'one to many' map.

`TableMap(java.util.Vector<java.lang.String> keys,
java.util.Vector<java.lang.String> values)`
  
          Initialise with a list of key-value pairs


| **Method Summary** | |
| --- | --- |
| `void` | `addKeyValue(java.lang.String key, java.lang.String value)`             Add a key/value set to the one to many map |
| `boolean` | `containsKey(java.lang.String key)` |
| `boolean` | `containsKeyValue(java.lang.String key, java.lang.String value)` |
| `boolean` | `containsValue(java.lang.String value)` |
| `void` | `deleteKeyValue(java.lang.String key, java.lang.String value)`             Delete a key-value pair from the one to many map |
| `java.util.Set<java.lang.String>` | `getKeys()` |
| `int` | `getKeyValuePairSize()`             Get the number of key-value pairs in the map |
| `java.util.Set<java.lang.String>` | `getKeyValues(java.lang.String key)` |
| `OneToManyMap<java.lang.String,java.lang.String>` | `getMap()`             Get the keys to values map |
| `OneToManyMap<java.lang.String,java.lang.String>` | `getReverseMap()`             Get the values to keys map |
| `java.util.Set<java.lang.String>` | `getValues()` |

**Methods inherited from class java.lang.Object**

`equals, getClass, hashCode, notify, notifyAll, toString, wait, wait, wait`

**Constructor Detail**

### TableMap

```
public TableMap()
```

:   Initialises the object with an empty 'one to many' map.

---


### TableMap

```
public TableMap(java.util.Vector<java.lang.String> keys,
                java.util.Vector<java.lang.String> values)
```

:   Initialise with a list of key-value pairs

    **Parameters:**: `keys` - the list of keys: `values` - the list of values (the value for each key in the keys list)


**Method Detail**

### addKeyValue

```
public void addKeyValue(java.lang.String key,
                        java.lang.String value)
```

:   Add a key/value set to the one to many map

    :   **Parameters:**: `key` -: `value` -

---


### containsKey

```
public boolean containsKey(java.lang.String key)
```

---


### containsKeyValue

```
public boolean containsKeyValue(java.lang.String key,
                                java.lang.String value)
```

---


### containsValue

```
public boolean containsValue(java.lang.String value)
```

---


### deleteKeyValue

```
public void deleteKeyValue(java.lang.String key,
                           java.lang.String value)
```

:   Delete a key-value pair from the one to many map

    :   **Parameters:**: `key` -: `value` -

---


### getKeys

```
public java.util.Set<java.lang.String> getKeys()
```

:   **Returns:**: the set of keys

---


### getKeyValuePairSize

```
public int getKeyValuePairSize()
```

:   Get the number of key-value pairs in the map

    :   **Returns:**: the number of key-value pairs in the map (each key can have more than one value)

---


### getKeyValues

```
public java.util.Set<java.lang.String> getKeyValues(java.lang.String key)
```

---


### getMap

```
public OneToManyMap<java.lang.String,java.lang.String> getMap()
```

:   Get the keys to values map

    :   **Returns:**: the map containing the data (each key and its values vector)

---


### getReverseMap

```
public OneToManyMap<java.lang.String,java.lang.String> getReverseMap()
```

:   Get the values to keys map

    :   **Returns:**: the map containing the data (each value and the vector of keys that have that value)

---


### getValues

```
public java.util.Set<java.lang.String> getValues()
```

:   **Returns:**: the set of values


---


|  |  |  |
| --- | --- | --- |
| **Overview** \| **Package** \| **Class** \| **Use** \| **Tree** \| **Deprecated** \| **Index** \| **Help** | |  |
| **PREV CLASS**   NEXT CLASS | **FRAMES**    **NO FRAMES**     **All Classes** |  |
| SUMMARY: NESTED \| FIELD \| CONSTR \| METHOD | DETAIL: FIELD \| CONSTR \| METHOD |  |


---
